# Exome QTL-seq maps monogenic locus and QTLs in barley

**DOI:** 10.1186/s12864-017-3511-2

**Published:** 2017-02-02

**Authors:** Hiroshi Hisano, Kazuki Sakamoto, Hiroki Takagi, Ryohei Terauchi, Kazuhiro Sato

**Affiliations:** 1 Institute of Plant Science and Resources, Okayama University, 2-20-1 Chuo, Kurashiki, Okayama 710-0046 Japan; 2 Iwate Biotechnology Research Center, Kitakami, Iwate 024-0003 Japan

**Keywords:** Exome sequencing, *Hordeum vulgare*, Kernel color, Mapping, Net blotch, QTL-seq

## Abstract

**Background:**

QTL-seq, in combination with bulked segregant analysis and next-generation sequencing (NGS), is used to identify loci in small plant genomes, but is technically challenging to perform in species with large genomes, such as barley. A combination of exome sequencing and QTL-seq (exome QTL-seq) was used to map the mono-factorial Mendelian locus black lemma and pericarp (*Blp*) and QTLs for resistance to net blotch disease, a common disease of barley caused by the fungus *Pyrenophora teres*, which segregated in a population of 100 doubled haploid barley lines.

**Methods:**

The provisional exome sequences were prepared by ordering the loci of expressed genes based on the genome information and concatenating genes with intervals of 200-bp spacer "N" for each chromosome. The QTL-seq pipeline was used to analyze short reads from the exome-captured library.

**Results:**

In this study, short NGS reads of bulked total DNA samples from segregants with extreme trait values were subjected to exome capture, and the resulting exome sequences were aligned to the reference genome. SNP allele frequencies were compared to identify the locations of genes/QTLs responsible for the trait value differences between lines. For both objective traits examined, exome QTL-seq identified the monogenic Mendelian locus and associated QTLs. These findings were validated using conventional mapping approaches.

**Conclusions:**

Exome QTL-seq broadens the utility of NGS-based gene/QTL mapping in organisms with large genomes.

**Electronic supplementary material:**

The online version of this article (doi:10.1186/s12864-017-3511-2) contains supplementary material, which is available to authorized users.

## Background

Bulked segregant analysis (BSA) has been combined with whole-genome sequencing (WGS) to rapidly identify causative nucleotide changes in a given mutant (MutMap [1]) and quantitative trait loci (QTLs) (QTL-seq [2]) in rice. Although these methods are suitable for studies of organisms with relatively small genomes (e.g., genome sizes of <1 Gbp), they have limited utility in crops with larger genomes due to the high cost of WGS and the difficulty of assembling large amounts of sequencing data.

Barley (*Hordeum vulgare* L.) is a diploid species with a genome size of 5.1 Gbp. A draft genome sequence of barley cv. Morex based on BAC fingerprinting, BAC-end sequencing, whole-genome shotgun sequencing, and RNA-seq data has been published [3]. The sequences obtained using these techniques were anchored to high-resolution genetic maps to infer the physical positions of the loci. To obtain gene model information, full-length cDNAs (FLcDNAs) derived from cv. Haruna Nijo [4, 5] were also mapped to the genome.

Exome capture is a standard technique used to sequence individuals of species with large genomes. Exome capture has been widely used in studies of humans, since the human exome, which represents only 1–2% of the total genome (3 Gbp), includes ca. 85% of the genetic variation responsible for hereditary human diseases [6]. By combining gene models and FLcDNAs information, Mascher et al. [7] developed an exome capture system to enrich for genic fragments from the barley genome. The resulting exome, estimated to be 61.6 Mbp, can readily be sequenced. The authors captured exomes from haplotypes of 20 wild and domesticated barley lines and one common wheat (*Triticum aestivum*) cultivar and mapped their reads onto the barley gene models. This analysis revealed a high level of sequence polymorphisms in the genic regions of the barley haplotypes examined. By combining captured exomes and mapping-by-sequencing, Mascher et al. [8] narrowed down a barley mutant gene to a single BAC contig scaffold.

Haruna Nijo is a Japanese malting barley cultivar that has been used for BAC library development [9] and whole-genome shotgun sequencing [10]. The wild barley (*H. vulgare* ssp. *spontaneum*) accession H602, which is distantly related to Haruna Nijo, was used to generate recombinant chromosome substitution lines (RCSLs) for genetic mapping [11] and a high-resolution transcript map [12]. The following key traits segregated in this mapping population: black and white lemma and pericarp color, which was determined by the putative gene *Blp* (black lemma and pericarp [13]); resistance and susceptibility to net blotch disease caused by the fungus *Pyrenophora teres* f. *teres*, [14]; and a seed dormancy QTL, which was designated *Qsd1* [15].

The black lemma and pericarp phenotype caused by *Blp* is a trait often found in landraces of Central Asia, Ethiopia, and the Himalayan area. The classic genetic study of this trait [16] revealed that it is inherited in a mono-factorial Mendelian fashion. The causative gene is located on the long arm of chromosome 5H, as revealed by genetic studies involving a cross between Haruna Nijo and H602 [17] and another population of Oregon Wolfe Barleys [18].

Net blotch is one of the main foliar diseases of barley worldwide [19]. Genetic studies suggest that multiple resistance factors control the plant’s reaction to the causal fungus, *P. teres* f. *teres* [20, 21]. Sato and Takeda [14] reported that H602 has the highest level of resistance among the barley germplasms examined, but most malting barleys, including Haruna Nijo, are susceptible to *P. teres*. The resistance QTLs were found to be located on chromosomes 3H and 6H, as revealed by a cross between the susceptible (sensitive) line ‘Hector’ and the resistant (insensitive) line ‘NDB 112’ [22].

In the current study, we performed next-generation sequencing (NGS)-based locus mapping of barley using an exome capture technique. Specifically, we modified the QTL-seq technique [2] for use in barley by employing a reference genome in which barley exomes are joined in the order of their linkage positions in the genome. To demonstrate the utility of this method, we mapped qualitative and quantitative traits with known locus positions in barley, e.g., *Blp* and susceptibility to *P. teres*, which segregate in the well-established doubled haploid mapping population of Haruna Nijo x H602.

## Methods

### Scoring of kernel color

A mapping population of 100 doubled haploid (DH) lines derived from a cross between Haruna Nijo and H602 carrying *blp* and *Blp*, respectively, (Additional file 1: Figure S1) [12] was used. All lines were planted in the experimental field of the Institute of Plant Science and Resources, Okayama University in Kurashiki, Japan and scored for kernel color at maturity. Among individuals in this population, 52 lines had a black lemma and pericarp (*Blp*) phenotype and 48 lines had a straw-white lemma and pericarp (*blp*) phenotype (Additional file 1: Figure S1).

### Scoring of net blotch resistance and QTL mapping

A single spore of *Pyrenophora teres* f. *teres* was isolated from an infected leaf of malting barley collected in the field at Chikugo, Fukuoka, Japan and prepared as an inoculum according to the culture method of Sato and Takeda [14]. Two-leaf stage seedlings were inoculated with *P. teres* in a growth chamber as described by Sato and Takeda [14], and the second leaves were scored on a scale of 1 (highly resistant) to 10 (highly susceptible) [23]. Two plants were tested per line, and the entire experiment was replicated twice. Based on the genotyping data of 1,116 SNPs identified in an oligonucleotide pooled assay of DH lines [11], linkage between SNP markers and QTLs responsible for reactions to *P. teres* was detected via the composite interval mapping procedure of QTL Cartographer v. 2.5 [24]. Significance of LOD (logarithm of odds) scores were calculated using a 1000 permutation test.

### Construction and sequencing of a bulked exome capture library

Genomic DNA was isolated from the fresh leaves of each DH line using a DNeasy Plant Mini Kit (QIAGEN, Hilden, Germany). The DNA concentration was measured with a Qubit® 2.0 fluorometer (Thermo Fisher Scientific, Waltham, MA, USA). DNA from each DH line was adjusted to a concentration of 20 ng/μl and mixed in an equal ratio to produce two bulked DNA pools per trait. For mapping of the *Blp* locus, 52 black lines and 48 white lines were pooled, and for net blotch resistance, 10 highly resistant and 10 susceptible lines were pooled.

For fragmentation of genome DNA, 1 μg of bulked DNA was sheared to approximately 200-bp fragments with an M220 Focused-ultrasonicator™ (Covaris, Woburn, MA, USA) in a 50 μl volume in a microTUBE AFA Fiber Screw-Cap vessel. A whole genome (WG) library barcoded by index sequences was constructed from the fragmented bulked DNA with a KAPA Library Preparation Kit (Kapa Biosystems, Wilmington, MA, USA) following the manufacturer’s protocol. Four libraries, including black and white bulks for *Blp* and resistant and susceptible bulks for net blotch resistance, were constructed. After evaluating the quality and size of the WG libraries in a Bioanalyzer (Agilent Technologies, Santa Clara, CA, USA) with an Agilent DNA 1000 Kit (Agilent Technologies), 1 μg of the WG library generated from two bulked samples (i.e., black and white for *Blp*; resistant and susceptible for net blotch resistance) was mixed. Fragments in the two mixed WG libraries harboring exon sequences were captured using the SeqCap EZ Library SR (Roche Diagnostics, Basel, Switzerland) designed for the barley genome [7], following the manufacturer’s protocol. The captured fragments (exome-captured library; EC library) were amplified and evaluated for quality and size in a Bioanalyzer with an Agilent DNA 1000 Kit. To obtain paired-end reads (150 bp × 2), the EC libraries were sequenced by MiSeq (Illumina, San Diego, CA, USA) with a MiSeq v2 Reagent Kit 300 Cycles (Illumina), following the manufacturer’s protocol. The short genomic reads obtained in this study were deposited at DDBJ-BioProject under accession number PRJDB4643.

### Sequence data analysis and generation of the SNP index

QTL-seq requires a step in which the reference sequence of one of the parents used for the cross is reconstructed. In the current study, provisional exome sequences (PESs, Additional file 2: Table S1) were first regenerated from published gene models [3] derived from RNA-seq data from cv. Morex and FLcDNAs from cv. Haruna Nijo. The PESs were prepared by ordering the loci of expressed genes (MLOC: 50.67 Mbp, 35,134 loci) based on genome information [3] and concatenating genes with intervals of 200-bp ‘N’ (where N is a spacer) for each chromosome. A set of 3.9-Gbp RNA-seq reads of Haruna Nijo ([10], Additional file 2: Table S2) were downloaded, and after low-quality sequences were trimmed using Trimmomatic [25], the remaining reads were mapped onto the PESs to construct the pseudo reference sequence (PRS). Nucleotides of the Morex PESs were replaced with those of the Haruna Nijo haplotype to generate the Haruna Nijo PRS.

The QTL-seq pipeline [2] was used to analyze short reads from the exome-captured library. The number of reads in each bulk was adjusted to the smaller number and used for the further analysis. After mapping the short reads to the PRS, the SNP index and ΔSNP index values were detected, and the average values were calculated by sliding window analysis. The “window size” was configured from 100 kbp to 1 Mbp, and “slide size” was set to 10 kbp. The SNP-calling filter “Coval” [26] was set to 6. Next, SNP positions with a SNP index of <0.3 were excluded, and a threshold of more than seven mismatches and a depth of fewer than four were eliminated, as these SNPs may be due to sequencing and/or alignment errors.

## Results

### Construction of the pseudo reference sequence

As a result of adding a reference sequence step to the QTL-seq pipeline, ca. 3.2 million Haruna Nijo RNA-seq reads were mapped onto the provisional exome sequences (PESs), and a total of 25,451 SNPs was detected between the RNA-seq reads and the PESs (Additional file 2: Table S3), in which the nucleotides from Morex were replaced with those from Haruna Nijo. The reconstructed pseudo reference sequence (PRS) of Haruna Nijo was used for further analysis.

### Mapping the Blp locus

Among the 100 DH lines, 52 lines had the black lemma and pericarp (*Blp*) phenotype and 48 had the straw-white lemma and pericarp﻿ (*blp*) phenotype. The segregation fitted a mono-factorial Mendelian ratio of 1:1 (χ^2^ = 0.16, *p* = 0.69, df = 1). Two sequence libraries were constructed using pooled DNA from the black and white lines, respectively. After two rounds of sequencing runs of mixed libraries from black and white bulks via Illumina MiSeq, ca. 31 million reads (amounting to 4.6 Gbp) and ca. 32 million reads (4.7 Gbp) were obtained for the black and white libraries, respectively (Additional file 2: Table S4). After applying the QTL-seq pipeline, 11,571,701 paired reads (ca. 2.314 Gbp) from the black library and 12,217,344 paired reads (ca. 2.443 Gbp) from the white library were mapped to the PRS (Additional file 2: Table S4). After quality filtration at a level of Coval = 6, we obtained 40,701 SNPs with ca. 3.6 million reads in the black bulk and 35,218 SNPs with ca. 3.4 million reads in the white bulk (Table 1). These SNPs were distributed throughout the genome (Table 1, Additional file 1: Figure S2).

**Table 1:** Number of aligned reads and detected SNPs for *Blp* revealed by exome QTL-seq

	Library^a^	1H	2H	3H	4H	5H	6H	7H	Total
No. of aligned reads	Black	452,145	612,949	550,473	360,920	600,028	457,033	573,584	3,607,132
	White	436,249	568,167	514,119	335,529	556,315	427,664	535,036	3,373,079
No. of detected SNPs	Black	6,745	6,791	5,806	3,540	6,064	5,848	5,907	40,701
	White	4,253	6,351	5,180	3,423	5,605	4,871	5,535	35,218

^a^Black and White indicate the color of both the lemma and the pericarp in each phenotypic bulk

**Fig. 1:**
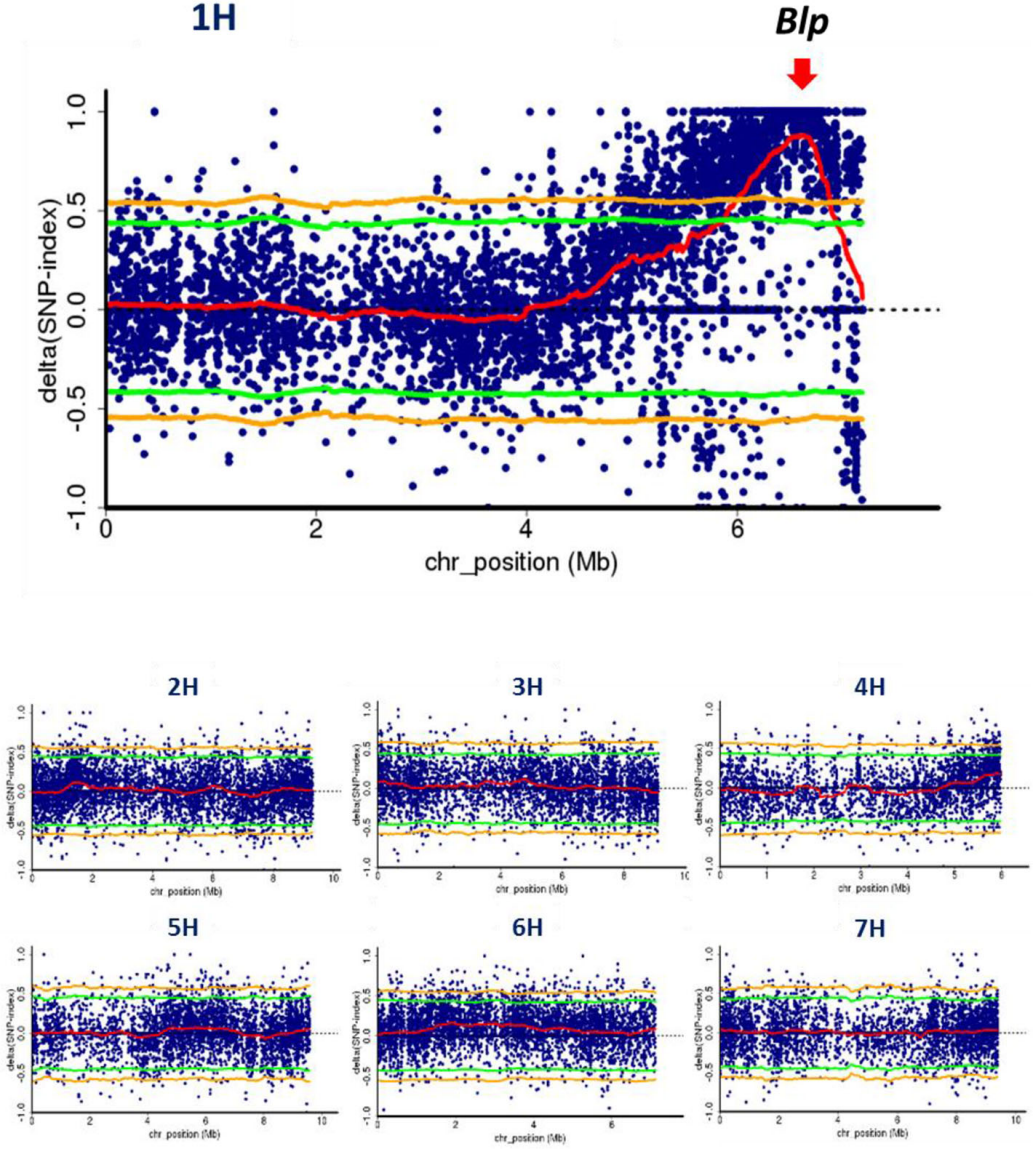
ΔSNP index plots of each chromosome generated by QTL-seq analysis for use in *Blp* mapping. Each chromosome contains loci of expressed genes (1H: 4,300; 2H: 5,582; 3H: 5,556; 4H: 3,647; 5H: 5,859; 6H: 4,307; and 7H: 5,883) concatenating with intervals of 200-bp ‘N’ as a spacer. The ΔSNP index was obtained by subtracting the *white* bulk SNP index from the *black* bulk SNP index. The thick *red* line represents the sliding window average of the ΔSNP index (window size, 500 kbp; slide size, 10 kbp). The *red* arrow indicates the position of *Blp* as detected by Hori et al. [17]. Statistical confidence intervals under the null hypothesis of no QTL are indicated by *yellow* (*p* < 0.01) and *green* (*p* < 0.05) lines

The ΔSNP index was obtained for each SNP between black (Additional file 1: Figure S3a) and white (Additional file 1: Figure S3b) bulks. These values were calculated by sliding window analysis and plotted onto the PRS, in which the genes were ordered based on their positions in the barley genome [3]. Figure 1 shows a plot of the ΔSNP index obtained after setting the sliding window size to 500 kbp, window increment size to 10 kbp, and Coval to 6. The ΔSNP index peaked above the 1% level of statistical significance on the long arm of chromosome 1H. There were no other significant peaks in the genome. After changing the sliding window size to 100 kbp or 1 Mbp, the same SNP index peak was still observed (Additional file 1: Figure S4).

### QTL analysis of net blotch resistance

**Fig. 2:**
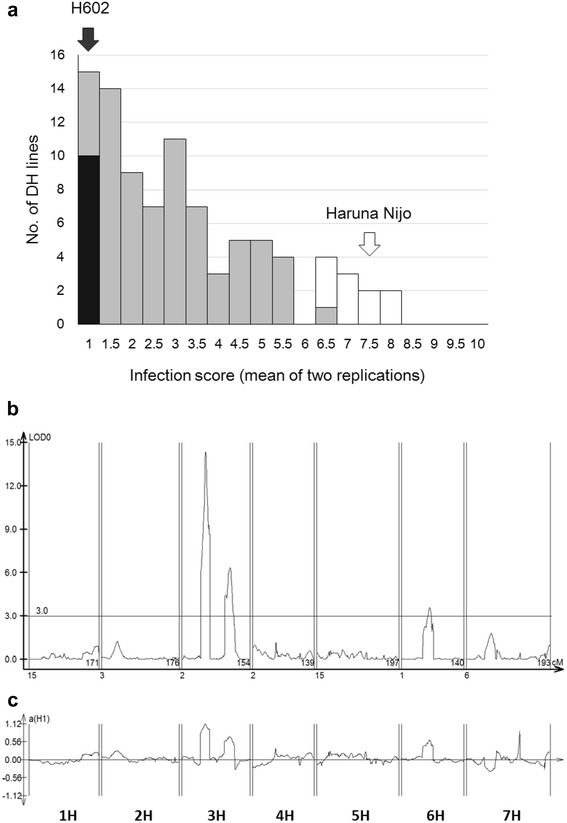
QTL analysis of net blotch resistance in a doubled haploid population derived from a cross between Haruna Nijo and H602. **a** Frequency distribution of average scores of two replications of the reaction to *P. teres* in doubled haploid lines. The average scores of Haruna Nijo and H602 are indicated by *white* and *black* arrows, respectively. *Black* and *white* bars indicate the number of lines used to produce the resistant and susceptible bulks, respectively, for QTL-seq analysis. **b** LOD scores for net blotch resistance and **c** additive effects. The markers are ordered following the genetic positions and the directions of chromosomes, which are oriented from the short arm of chromosome 1H on the left side to the long arm of chromosome 7H along the horizontal axis

Based on the reaction scores of the doubled haploid lines (Fig. 2a) and the genotyping data of 1,116 SNP markers [11], we identified QTLs associated with the *P. teres* resistance trait. Figure 2b shows the positions of QTLs and Fig. 2c shows their additive values. Two QTLs were detected on chromosome 3H (42.9–63.1 cM, LOD = 14.2, *r* ^2^ = 0.27; 96.6–116.6 cM, LOD = 6.6, *r* ^2^ = 0.10) and another QTL was detected on chromosome 6H (62.4–63.9 cM, LOD = 4.2, *r* ^2^ = 0.06). All of the peaks could be explained by the presence of H602 alleles for resistance to *P. teres* (Fig. 2c).

### Mapping net blotch resistance

We investigated the *P. teres* reaction scores of 100 doubled haploid lines. The segregation of these lines based on net blotch resistance is multimodal and does not follow the normal distribution, suggesting that this trait undergoes oligogenic inheritance. We confirmed this possibility by QTL analysis (Fig. 2b and c). We selected 10 net blotch resistant and 10 susceptible lines to produce two bulk DNA samples for exome sequence library construction (see also Additional file 1: Figure S5 for the resistance reactions of the parents). After one round of sequencing of pooled resistant and susceptible bulk libraries via Illumina MiSeq, DNA sequences of ca. 22 million reads (3.2 Gbp) and ca. 21 million reads (3.1 Gbp) were obtained from the resistant (R) and susceptible (S) bulk libraries, respectively (Additional file 2: Table S5). After applying the QTL-seq pipeline, 8,510,990 paired reads (ca. 1.702 Gbp) and 8,138,104 paired reads (ca. 1.628 Gbp) from the R and S bulk libraries, respectively, were mapped to the PRS (Additional file 2: Table S5). After quality filtration at Coval = 6, we obtained 40,110 SNPs with ca. 2.3 million reads in the R bulk and 32,837 SNPs with ca. 2.4 million reads in the S bulk (Table 2), which were distributed throughout the genome (Table 2, Additional file 1: Figure S6); however, there were twice as many SNPs on both chromosomes 3H and 6H in the R bulk than in the S bulk.

**Table 2:** Number of aligned reads and detected SNPs for net blotch resistance revealed by exome QTL-seq

	Library^a^	1H	2H	3H	4H	5H	6H	7H	Total
No. of aligned reads	R	289,281	385,073	340,122	225,445	378,332	285,861	360,933	2,265,047
	S	304,488	398,271	366,993	234,005	391,138	304,305	377,452	2,376,652
No. of detected SNPs	R	5,024	6,125	8,167	2,752	5,694	7,171	5,177	40,110
	S	4,048	6,574	4,212	2,805	6,328	3,506	5,364	32,837

^a^R and S indicate bulks of lines showing resistance and susceptibility to *P. teres*, respectively

**Fig. 3:**
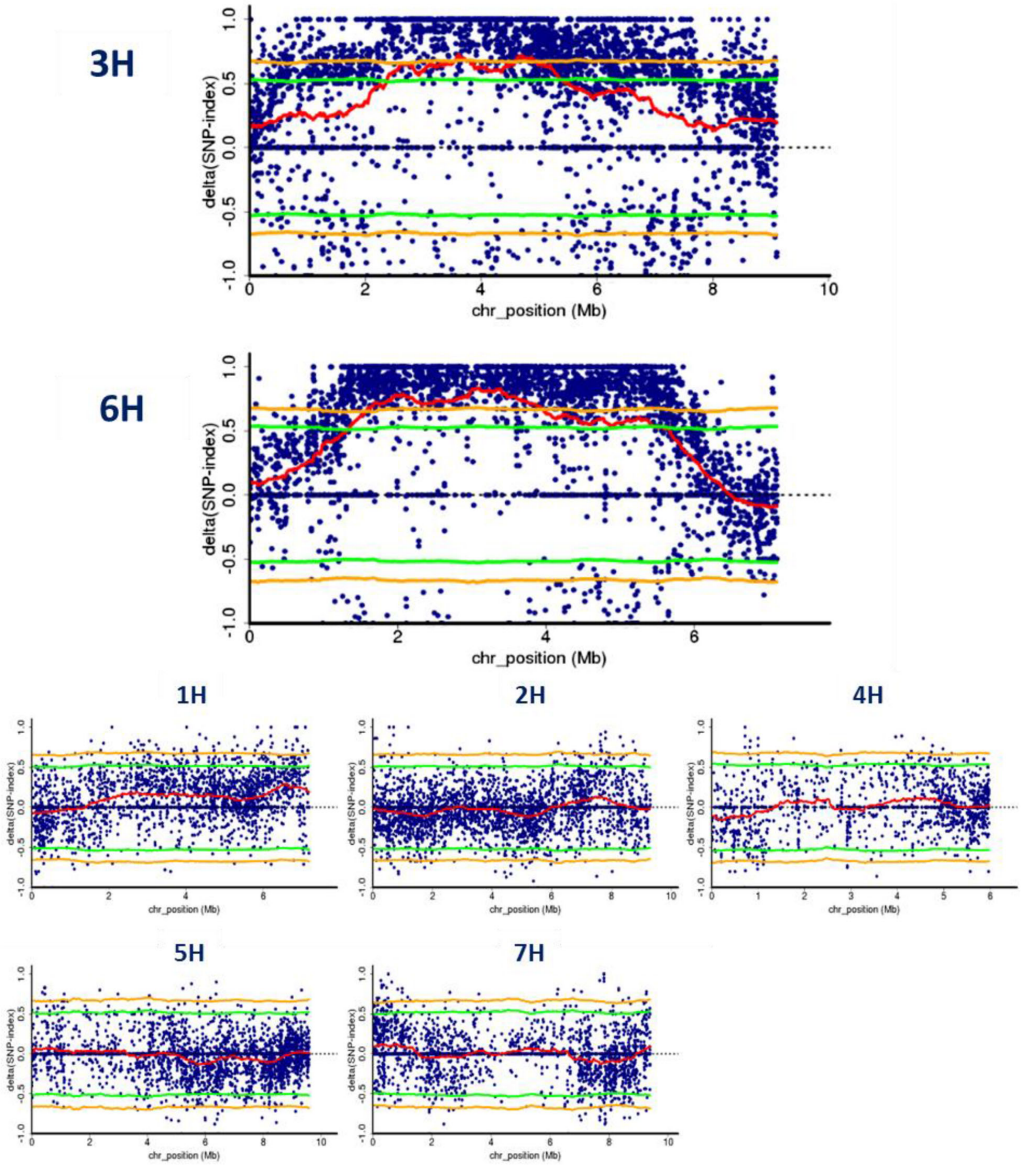
ΔSNP index plots for each chromosome generated by QTL-seq analysis for use in mapping of the net blotch resistance trait. Each chromosome contains loci of expressed genes (1H: 4,300; 2H: 5,582; 3H: 5,556; 4H: 3,647; 5H: 5,859; 6H: 4,307 and 7H: 5,883) concatenating with intervals of 200-bp ‘N’ as a spacer. The ΔSNP index was obtained by subtracting the S bulk SNP index from the R bulk SNP index. The thick *red* line represents the sliding window average of the ΔSNP index (window size, 750 kbp; slide size, 10 kbp). Statistical confidence intervals under the null hypothesis of no QTL are shown by *yellow* (*p* < 0.01) and *green* (*p* < 0.05) lines

We calculated the ΔSNP index scores between the R (Additional file 1: Figure S7a) and S (Additional file 1: Figure S7b) bulks and plotted these scores in the same manner as for *Blp* mapping. Figure 3 shows the ΔSNP index scores calculated with a sliding window size of 750 kbp, slide size of 10 kbp, and Coval of 6. The ΔSNP index peaked above the 1% level of statistical significance only on chromosomes 3H and 6H. We modified the window size to 500 kbp and 1 Mbp, which produced ΔSNP indices that were lower than those calculated using a window size of 750 kbp (Additional file 1: Figure S8).

## Discussion

Linkage mapping is used to detect markers that are inherited in a bi-parental segregating population and to arrange the markers on the map based on the frequency of crossing-over. Bulked segregant analysis (BSA) is a linkage mapping technique used to identify DNA markers linked to a particular locus. In this method, two bulked DNA samples are developed from a phenotypically segregating population, whose members are screened for DNA marker polymorphisms. Michelmore et al. [27] showed that markers can be reliably identified within a 25-cM window on either side of the targeted locus. MutMap [1] and QTL-seq [2] are essentially the same as BSA, but in these techniques, markers are generated by high-throughput sequencing of pooled DNA, and a large number of SNPs are mapped onto the genome. In QTL-seq, the mapping resolution depends on the number of individuals in the bulk [2], and the redundancy of sequence reads, including efficient SNPs, depends on the number of individuals examined. If there are sufficient numbers of individuals in the bulks, it is possible to identify the SNPs associated with the trait. To acquire a higher rate of redundancy of reads, it is useful to analyze a plant with a relatively small genome. Moreover, analyzing a plant with a large genome requires the use of high-speed computers with advanced processing capabilities. Thus, it is currently difficult to perform QTL-seq on plants with large genomes.

Exome capture is a standard technique for sequencing individuals of species with large genomes, especially in human and mouse. Only one exome capture system is available for barley [7]. We cannot evaluate the genome coverage of these exome sequences, because the complete genome sequence is not yet available for barley. However, the number of sequence reads in the barley gene models identified in the current study was sufficient to reconstruct the PRS in the QTL-seq (Additional file 1: Figure S2) and to estimate the SNP index (Figs. 1 and 3). These loci were concatenated to 200 Ns in the virtually developed chromosome sequence, which does not represent the true distance in the genome. Thus, the SNP index around the target locus may reflect a slightly modified linkage relationship, although each SNP index is not influenced by the concatenation of the locus to Ns.

*Blp* is a simple Mendelian trait that was used as a phenotypic marker on a classical linkage map [13]. In this study, we chose this trait as a model to estimate the efficiency of our mapping strategy. We used all *Blp* (52) and *blp* (48) individuals in a pool of 100 doubled haploid lines to maximize the mapping resolution. The expected redundancies of reads in the library were 28.0 in *Blp* and 30.0 in *blp*, which are fewer than the number of individuals per library (Additional file 1: Figure S2). Takagi et al. [2] suggested that the number of individuals used for QTL mapping in rice (*Oryza sativa*) by QTL-seq can be as low as 15% of those used for conventional QTL mapping by obtaining a higher read depth. Mascher et al. [7] used exome capture in barley to map an induced mutation in an F_2_ mapping population from a cross between cv. Barke and a mutant in the cv. Saale background. The authors used 18 mutant and 30 wild-type individuals to map the trait. Although we used more individuals in the current study, the mapping resolution can be further improved by increasing the sequence redundancy.

The net blotch resistance trait was used in this study to demonstrate the mapping of multiple loci controlling a single trait via sequencing analysis of bulks. Net blotch resistance has been identified as a quantitatively inherited trait, although classical linkage studies, including trisomic series analysis [28], have revealed resistance factors on chromosomes [19]. Liu et al. [22] identified net blotch resistance QTLs on chromosomes 3H and 6H based on a cross between the sensitive/susceptible cultivar ‘Hector’ and the insensitive/resistant line ‘NDB 112’, which correspond to the QTLs identified in the cross between Haruna Nijo and H602 in the current study (Fig. 2b and c). The QTLs on both chromosomes are located near centromeres, where crossing over is suppressed. Our interval mapping suggested that multiple QTLs are located on chromosome 3H. We cannot determine if these QTLs are distinct or the same; however, crossing overs between the QTL positions on chromosome 3H may help to establish whether multiple QTLs are present or absent. However, since multiple QTLs segregated in the population, only a limited number of individuals (10 for each) showing extreme phenotypes could be applied in this analysis. The results indicate the importance of having a higher number of individuals in the bulk, which would increase the mapping resolution by including more crossing over events when performing mapping analysis of multiple loci.

The mapping resolution of *Blp* and of the net blotch resistance QTLs obtained in the current study was not high enough to enable a comparison with results in rice [1, 2]. The mapping window sizes of *Blp* and net blotch resistance were 500 kbp and 750 kbp, respectively, which were much larger than those used for rice [2], and may blunt the peaks in the resulting maps. Furthermore, the lack of complete barley genome information complicated the mapping. The physical positions of BAC contigs were determined based on the genetic mapping of markers in a population of several hundred individuals [3]. Since multiple loci are located on a BAC contig with a single physical position, we could not estimate the order of loci on the same contig. Therefore, more complete genome information for barley must be obtained before high-resolution mapping of this plant can be conducted by exome QTL-seq.

## Conclusions

We generated a large number of SNP markers in barley by exome QTL-seq. Since these SNPs were localized to known loci on the barley genome, these markers can readily be used for map-based cloning of various loci. Thus, exome QTL-seq in barley provides opportunities not only for the direct mapping of a trait onto the genome, but also for generating markers that can be used to narrow down the position of a particular locus in the genome. For this purpose, the mapping resolution of exome QTL-seq in barley should be increased by using a larger number of individuals in the bulk and a higher redundancy of reads.

## Additional files

Additional file 1: Figure S1. Kernel color in the haploid mapping population. **Figure S2.** Depth of the mapped reads on the PRS in the exome-captured QTL-seq analysis for *Blp* mapping. **Figure S3.** Plots showing the SNP index of each chromosome generated by exome-captured QTL-seq analysis for *Blp* mapping in barley. **Figure S4.** Plots of the ΔSNP index of chromosome 1H generated by exome-captured QTL-seq analysis for Blp mapping. **Figure S5.** Infected leaf phenotypes. **Figure S6.** Depth of the mapped reads on PRS in the exome-captured QTL-seq analysis for net blotch resistance. **Figure S7.** Plots of the SNP index of each chromosome generated by exome-captured QTL-seq analysis for mapping of net blotch resistance. **Figure S8.** Plots of the ΔSNP index of chromosome 3H (left) and 6H (right) generated by exome-captured QTL-seq analysis for net blotch resistance. (DOCX 5291 kb)
Additional file 2: Table S1. Provisional exome sequences (PESs) based on Morex loci [3]. **Table S2.** RNA-seq data of Haruna Nijo used to restructure the pseudo reference sequence (PRS) in the QTL-seq analysis. **Table S3.** Number of aligned RNA-seq reads with PESs and detected SNPs against PES. **Table S4.** Number of reads and sequences used and mapped in the QTL-seq analysis of *Blp*. **Table S5.** Number of reads and sequences used and mapped in the QTL-seq analysis of net blotch resistance genes. (DOCX 32 kb)

## Abbreviations

BAC: Bacterial artificial chromosome
NGS: Next generation sequencing
QTL: Quantitative trait loci
SNP: Single nucleotide polymorphism
