# Supplementary material for: Exome QTL-seq maps monogenic locus and QTLs in barley
Source: BMC Genomics. 2017 Feb 2;18:125. doi: 10.1186/s12864-017-3511-2 (PMC5288901; doi:10.1186/s12864-017-3511-2)
Supplement: Additional file 1: Figure S1. — Kernel color in the haploid mapping population. Figure S2. Depth of the mapped reads on the PRS in the exome-captured QTL-seq analysis for Blp mapping. Figure S3. Plots showing the SNP index of each chromosome generated by exome-captured QTL-seq analysis for Blp mapping in barley. Figure S4. Plots of the ΔSNP index of chromosome 1H generated by exome-captured QTL-seq analysis for Blp mapping. Figure S5. Infected leaf phenotypes. Figure S6. Depth of the mapped reads on PRS in the exome-captured QTL-seq analysis for net blotch resistance. Figure S7. Plots of the SNP index of each chromosome generated by exome-captured QTL-seq analysis for mapping of net blotch resistance. Figure S8. Plots of the ΔSNP index of chromosome 3H (left) and 6H (right) generated by exome-captured QTL-seq analysis for net blotch resistance. (DOCX 5291 kb) [file 12864_2017_3511_MOESM1_ESM.docx]

**Supplemental Figures**

Figure S1


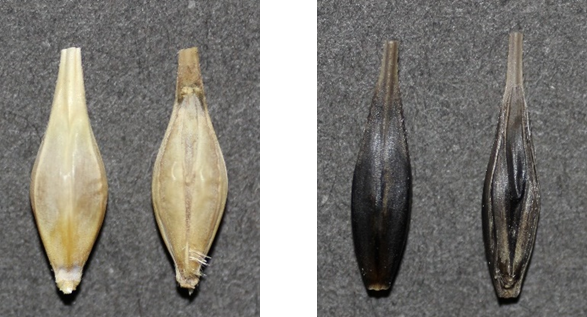


**Figure S1** Kernel color in the haploid mapping population.

Haruna Nijo (left panel, *blp*) has straw-white kernels and H602 (right panel, *Blp*) has black kernels. These lines were used as parents for developing a doubled haploid mapping population.

Figure S2


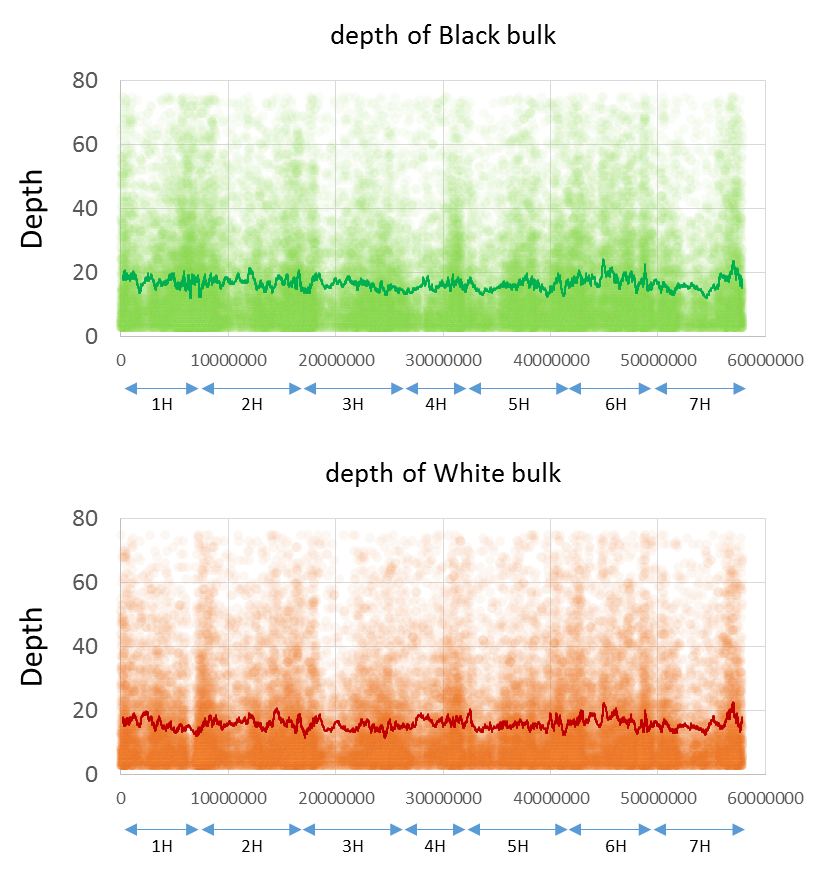


**Figure S2** Depth of the mapped reads on the PRS in the exome-captured QTL-seq analysis for *Blp* mapping.

Thick line indicates average value of depth drawn by the moving average of 255 SNP indices. Upper panel, depth of black bulk; Lower panel, depth of white bulk.

Figure S3 (a)


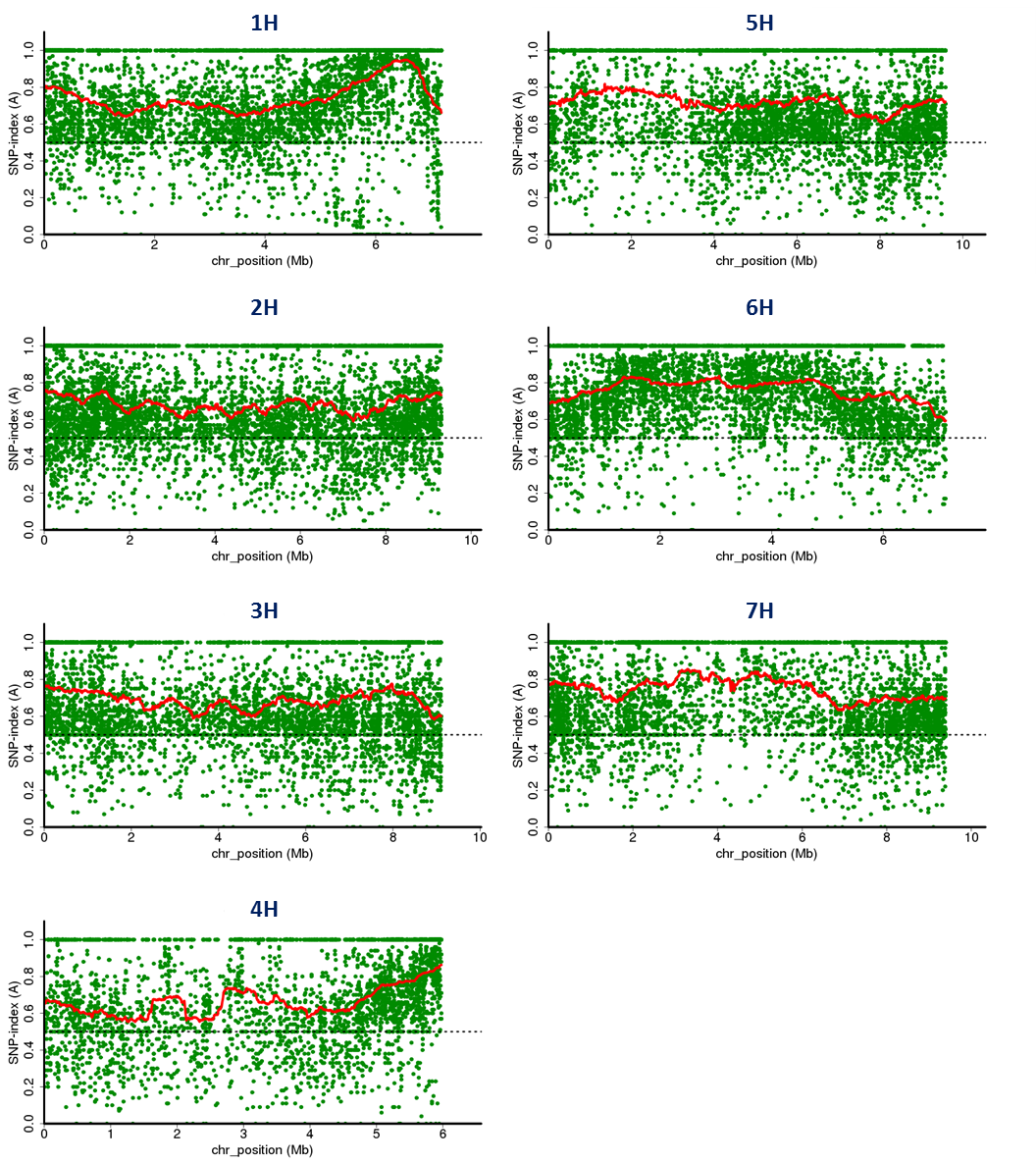


Figure S3 (b)


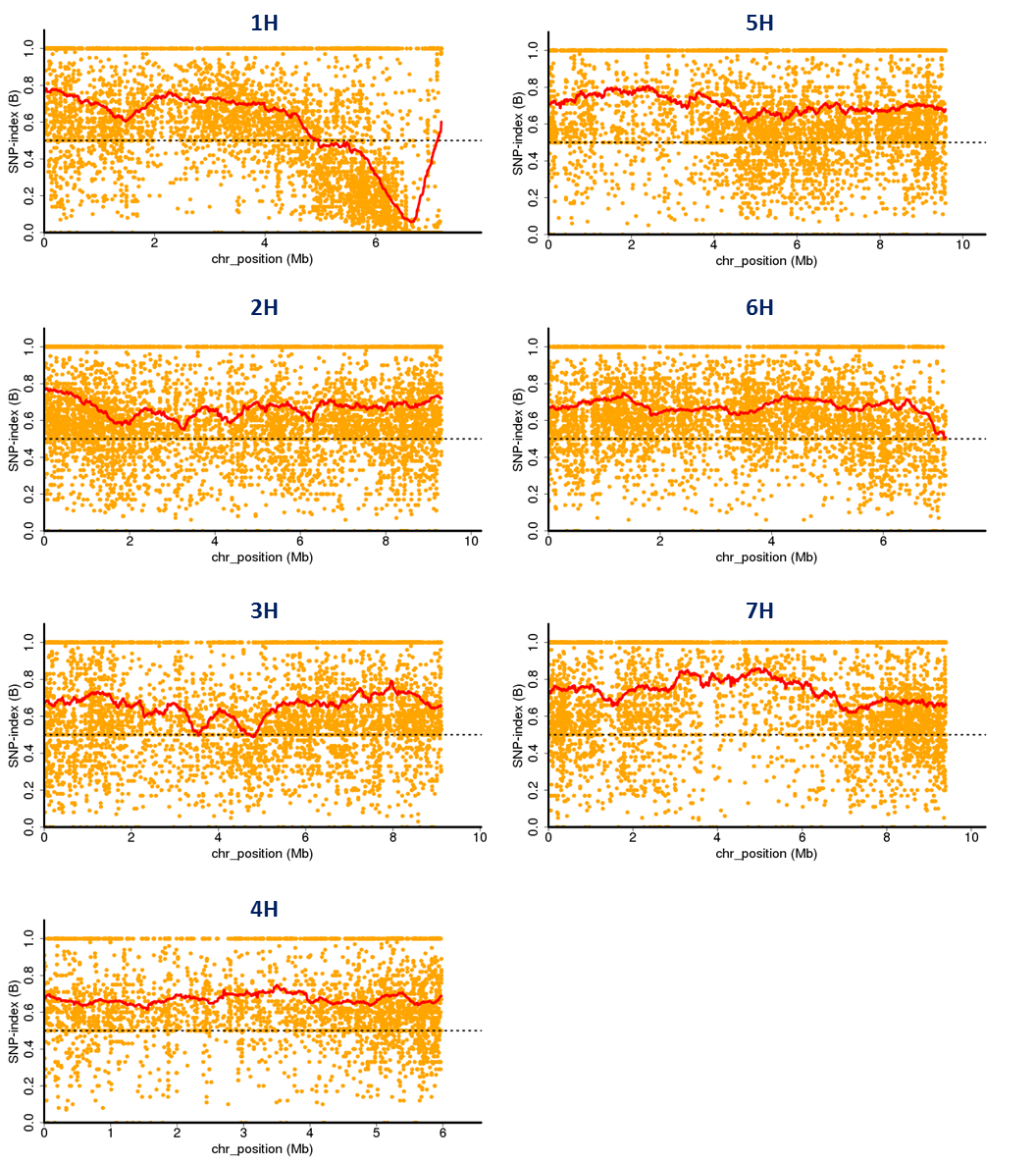


**Figure S3** Plots showing the SNP index of each chromosome generated by exome-captured QTL-seq analysis for *Blp* mapping in barley.

Each chromosome contains loci of expressed genes (1H: 4,300; 2H: 5,582; 3H: 5,556; 4H: 3,647; 5H: 5,859; 6H: 4,307 and 7H: 5,883) concatenating with intervals of 200-bp ‘N’ as a spacer. Green (a) and orange (b) dots show the SNP index of the black (a) and white (b) bulk, respectively. Red line represents the sliding window average of the SNP index (window size: 500 kbp; slide size: 10 kbp).

Figure S4


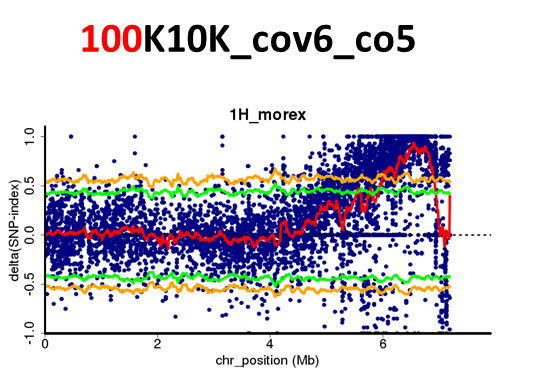


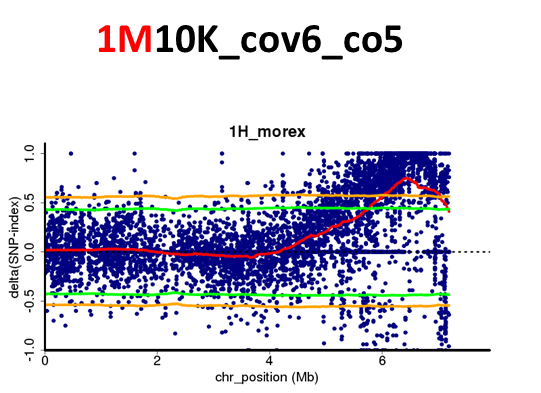


**Figure S4** Plots of the ΔSNP index of chromosome 1H generated by exome-captured QTL-seq analysis for *Blp* mapping.

Chromosome 1H contains 4,300 loci of expressed genes concatenating with intervals of 200-bp ‘N’ as a spacer. The ΔSNP index was obtained by subtracting the white bulk SNP index from the black one. Red thick line represents the sliding window average of the ΔSNP index. Window sizes were 100 kbp (upper panel) and 1 Mbp (lower panel) with a 10-kbp slide size. Confidence intervals under the null hypothesis of no QTL are indicated by the orange (*p*<0.01) and green (*p*<0.05) lines.

Figure S5


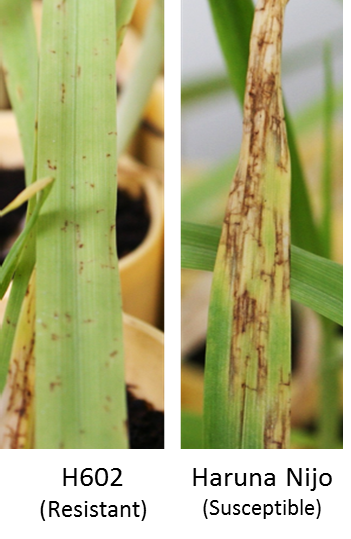


**Figure S5** Infected leaf phenotypes.

Lesions of H602 (left, resistant) and Haruna Nijo (right, susceptible) leaves 14 days after infection with *P. teres* were photographed.

Fig, S6


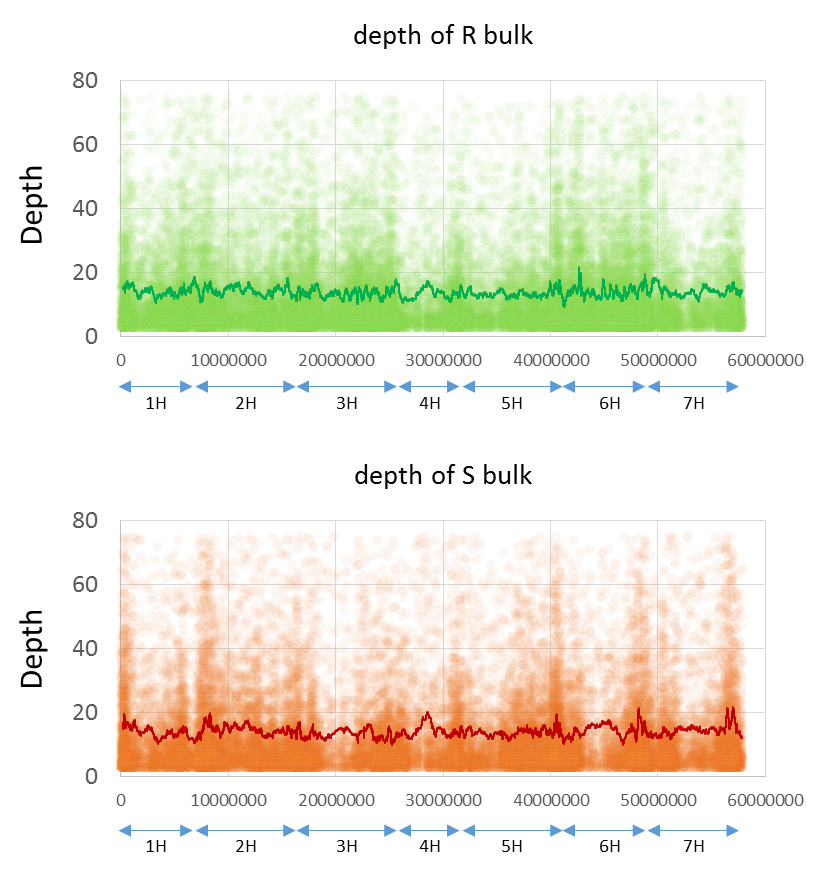


**Figure S6** Depth of the mapped reads on PRS in the exome-captured QTL-seq analysis for net blotch resistance.

Thick line indicates average value of depth drawn by the moving average of 255 SNP indices. Upper: depth of R (resistant) bulk. Lower: depth of S (susceptible) bulk.

Figure S7 (a)


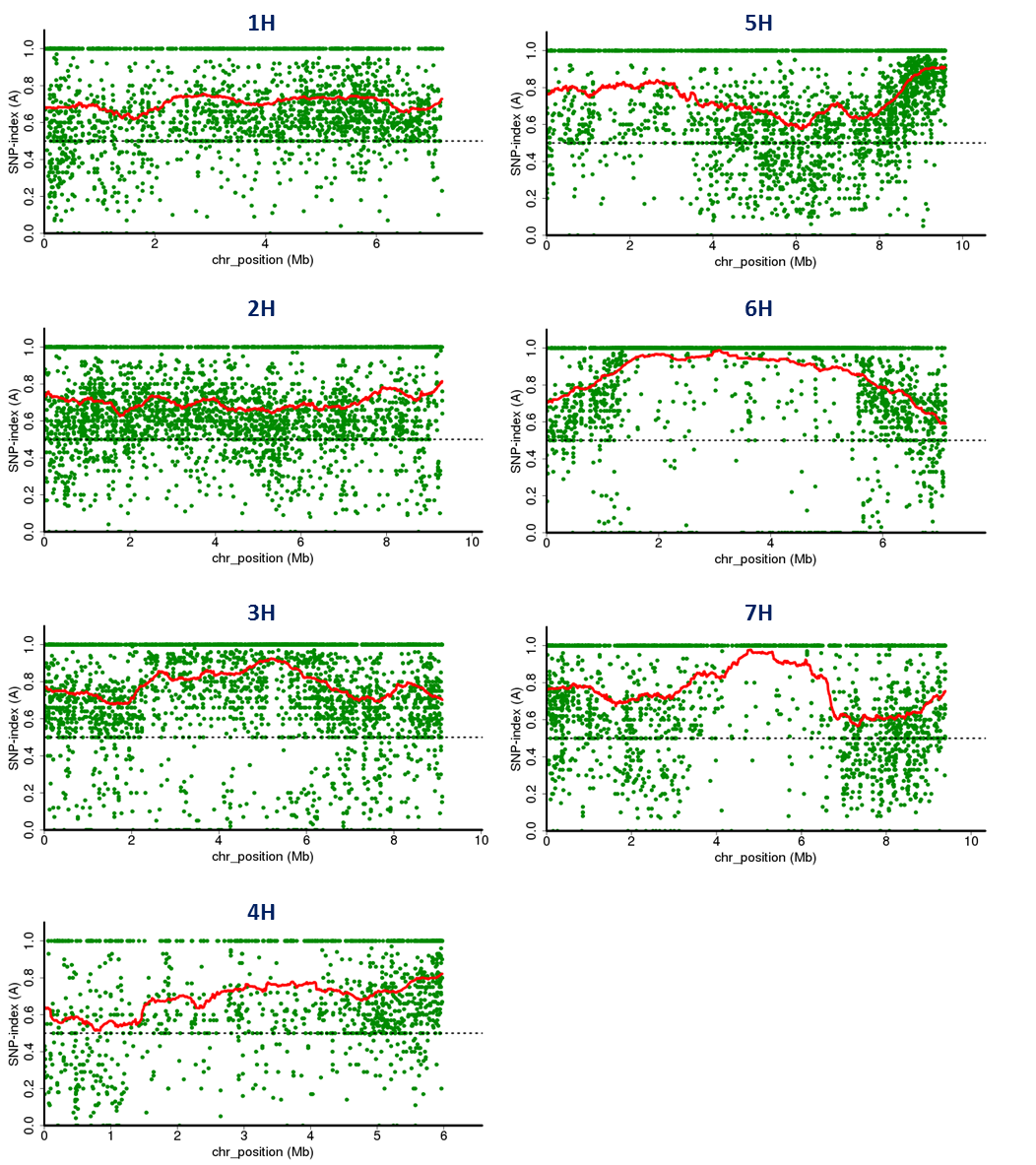


Figure S7 (b)


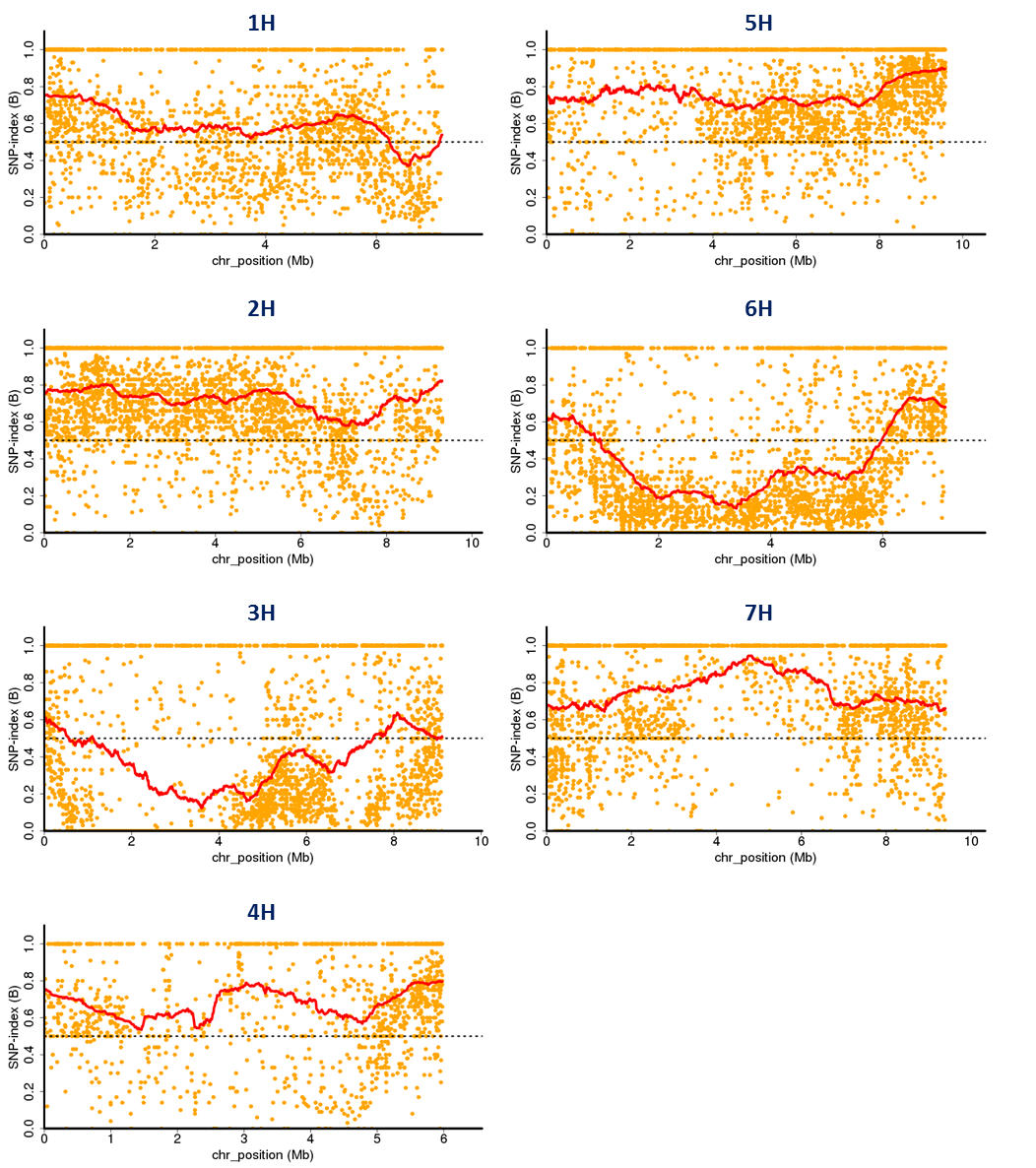


**Figure S7** Plots of the SNP index of each chromosome generated by exome-captured QTL-seq analysis for mapping of net blotch resistance.

Each chromosome contains loci of expressed genes (1H: 4,300; 2H: 5,582; 3H: 5,556; 4H: 3,647; 5H: 5,859; 6H: 4,307 and 7H: 5,883) concatenating with intervals of 200-bp ‘N’ as a spacer. Green (a) and orange (b) dots show the SNP index of the resistant (a) and susceptible (b) bulk, respectively. Red line represents the sliding window average of the SNP index (window size: 750 kbp; slide size: 10 kbp).

Figure S8


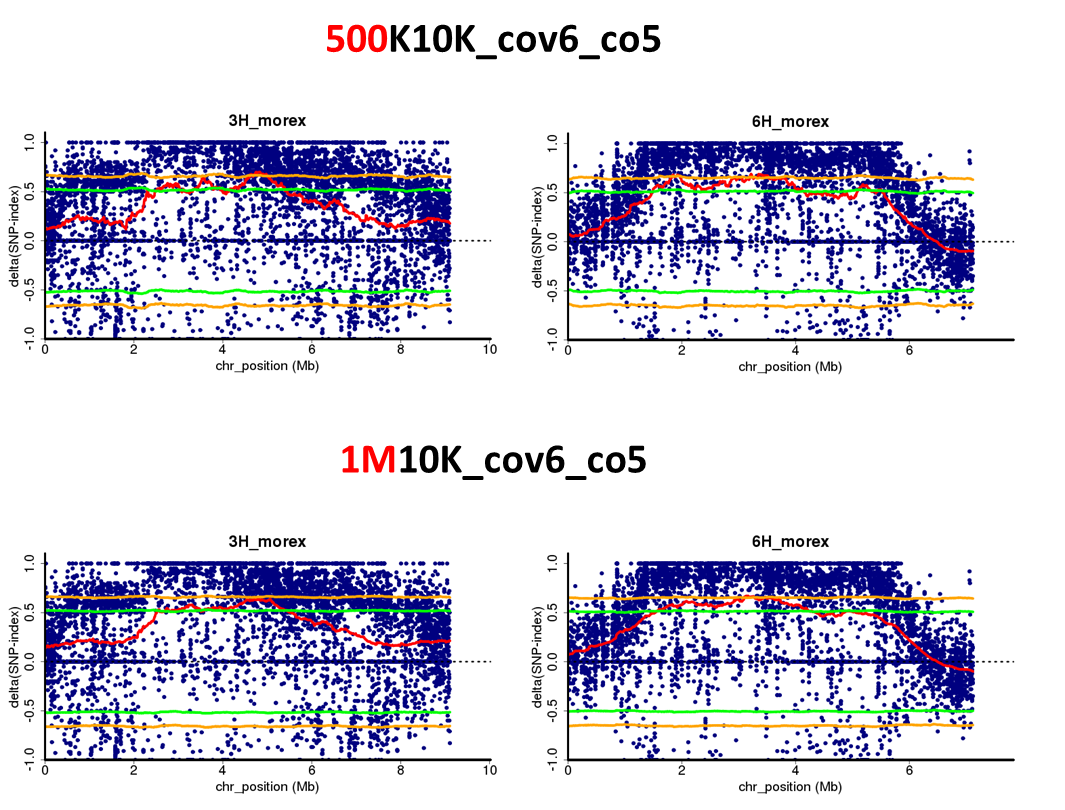


**Figure S8** Plots of the ΔSNP index of chromosome 3H (left) and 6H (right) generated by exome-captured QTL-seq analysis for net blotch resistance.

Each chromosome contains loci of expressed genes (3H: 5,556; and 6H: 4,307) concatenating with intervals of 200-bp ‘N’ as a spacer. The ΔSNP index was obtained by subtracting the white bulk SNP index from the black one. Red thick line represents the sliding window average of the ΔSNP index. Window size was 500 kbp (upper panels) and 1 Mbp (lower panels) with 10 kbp of slide size. Confidence intervals under the null hypothesis of no QTL are indicated by orange (*p*<0.01) and green (*p*<0.05) lines.
