# Supplementary material for: Exome QTL-seq maps monogenic locus and QTLs in barley
Source: BMC Genomics. 2017 Feb 2;18:125. doi: 10.1186/s12864-017-3511-2 (PMC5288901; doi:10.1186/s12864-017-3511-2)
Supplement: Additional file 2: Table S1. — Provisional exome sequences (PESs) based on Morex loci [3]. Table S2. RNA-seq data of Haruna Nijo used to restructure the pseudo reference sequence (PRS) in the QTL-seq analysis. Table S3. Number of aligned RNA-seq reads with PESs and detected SNPs against PES. Table S4. Number of reads and sequences used and mapped in the QTL-seq analysis of Blp. Table S5. Number of reads and sequences used and mapped in the QTL-seq analysis of net blotch resistance genes. (DOCX 32 kb) [file 12864_2017_3511_MOESM2_ESM.docx]

**Supplemental Tables**

| **Table S1** Provisional exome sequences (PES) based on gene models (IBSC 2012) | | | | | | | | |
| --- | --- | --- | --- | --- | --- | --- | --- | --- |
| Chromosome | 1H | 2H | 3H | 4H | 5H | 6H | 7H | Total |
| Length (Mbp) | 6.33 | 8.19 | 8.00 | 5.25 | 8.42 | 6.26 | 8.22 | 50.67 |
| # of loci | 4,300 | 5,582 | 5,556 | 3,647 | 5,859 | 4,307 | 5,883 | 35,134 |

| **Table S2** RNA-seq data of Haruna Nijo used to restructure the pseudo reference sequence (PRS) in the QTL-seq analysis | | | | | | | | | | | | |
| --- | --- | --- | --- | --- | --- | --- | --- | --- | --- | --- | --- | --- |
|  | Original ^b^ | | | |  | q30p90 ^c^ | | | |  | Paired ^d^ | |
| Tissue ^a^ | # reads | | Gbp | |  | # reads | | Gbp | |  | # reads | Gbp |
| spike_S2R1 | 4,362,456 | | 1.199 | |  | 3,676,309 | | 1.003 | |  | 4,146,724 | 1.088 |
| spike_S2R2 | 4,366,256 | | 1.207 | |  | 2,096,172 | | 0.549 | |  |  |  |
| root_S5R1 | 4,072,124 | | 1.191 | |  | 2,903,160 | | 0.846 | |  | 2,917,394 | 0.830 |
| root_S5R2 | 4,072,124 | | 1.192 | |  | 1,486,530 | | 0.422 | |  |  |  |
| seed_S2R1 | 7,176,768 | | 1.494 | |  | 5,259,843 | | 1.030 | |  | 7,098,348 | 1.244 |
| seed_S2R2 | 7,176,768 | | 1.542 | |  | 3,682,951 | | 0.643 | |  |  |  |
| shoot_S4R1 | 5,103,779 | | 1.514 | |  | 3,481,653 | | 1.032 | |  | 2,597,980 | 0.756 |
| shoot_S4R2 | 5,103,779 | | 1.515 | |  | 1,320,261 | | 0.383 | |  |  |  |
| total |  |  |  |  |  |  |  |  |  |  | 16,760,446 | 3.918 |
| ^a^ Tissue used for RNA-seq analysis. | | | | | | | | | | | | |
| ^b^ Data entered in the QTL-seq pipeline. | | | | | | | | | | | | |
| ^c^ Reads after filtering at q30 and p90. | | | | | | | | | | | | |
| ^d^ Reads mapped on PES. | | | | | | | | | | | | |

| **Table S3** Number of aligned RNA-seq reads with PESs and detected SNPs against PESs | | | | | | | | |
| --- | --- | --- | --- | --- | --- | --- | --- | --- |
| Chromosome | 1H | 2H | 3H | 4H | 5H | 6H | 7H | Total |
| # of reads | 420,100 | 545,924 | 472,824 | 375,835 | 544,733 | 388,550 | 442,951 | 3,190,917 |
| # of SNPs* | 3,184 | 4,770 | 3,857 | 2,193 | 4,573 | 2,767 | 4,107 | 25,451 |
| *Including In/Dels | | | | | | | | |

| **Table S4** Number of reads and sequences used and mapped in the QTL-seq analysis of *Blp* | | | | | | | | | | | | |
| --- | --- | --- | --- | --- | --- | --- | --- | --- | --- | --- | --- | --- |
|  | Original ^b^ | | | |  | q30p90 ^c^ | | | |  | Paired ^d^ | |
| Library ^a^ | # of reads | | Gbp | |  | # reads | | Gbp | |  | # reads | Gbp |
| Black_S1R1 | 6,773,918 | | 1.008 | |  | 6,206,783 | | 0.924 | |  | 5,328,989 | 1.066 |
| Black_S1R2 | 8,309,517 | | 1.237 | |  | 6,710,180 | | 0.998 | |  |  |  |
| Black2_S1R1 | 7,919,471 | | 1.179 | |  | 7,135,013 | | 1.062 | |  | 6,242,712 | 1.249 |
| Black2_S1R2 | 7,919,471 | | 1.179 | |  | 6,431,654 | | 0.956 | |  |  |  |
| total |  |  |  |  |  |  |  |  |  |  | 11,571,701 | 2.314 |
| White_S2R1 | 7,339,476 | | 1.084 | |  | 6,726,558 | | 0.993 | |  | 5,777,130 | 1.155 |
| White_S2R2 | 8,418,830 | | 1.243 | |  | 6,781,961 | | 1.000 | |  |  |  |
| White2_S2R1 | 8,037,076 | | 1.187 | |  | 7,245,688 | | 1.070 | |  | 6,440,214 | 1.288 |
| White2_S2R2 | 8,037,076 | | 1.187 | |  | 6,625,522 | | 0.977 | |  |  |  |
| total |  | |  | |  |  | |  | |  | 12,217,344 | 2.443 |
| ^a^ Black and White indicate the color of lemma and pericarp in each phenotypic bulk. | | | | | | | | | | | | |
| ^b^ The data put in QTL-seq pipeline. | | | | | | | | | | | | |
| ^c^ The reads after filtering at q30 and p90. | | | | | | | | | | | | |
| ^d^ The paired reads mapped on PRS. | | | | | | | | | | | | |

| **Table S5** Number of reads and sequences used and mapped in the QTL-seq analysis of net blotch resistance loci | | | | | | | | |
| --- | --- | --- | --- | --- | --- | --- | --- | --- |
|  | Original ^b^ | |  | q30p90 ^c^ | |  | Paired ^d^ | |
| Library ^a^ | # reads | Gbp |  | # reads | Gbp |  | # reads | Gbp |
| R-bulk_S1R1 | 10,804,652 | 1.604 |  | 9,419,488 | 1.398 |  | 8,510,990 | 1.702 |
| R-bulk_S1R2 | 10,804,652 | 1.604 |  | 8,826,594 | 1.309 |  |  |  |
| S-bulk_S2R1 | 10,360,477 | 1.544 |  | 9,034,851 | 1.346 |  | 8,138,104 | 1.628 |
| S-bulk_S2R2 | 10,360,477 | 1.544 |  | 8,411,573 | 1.252 |  |  |  |
| ^a^ R- and S-bulk indicate the bulks of lines showing resistance and susceptibility to *P. teres*. | | | | | | | | |
| ^b^ Data entered in the QTL-seq pipeline. | | | | | | | | |
| ^c^ Reads after filtering at q30 and p90. | | | | | | | | |
| ^d^ Paired reads mapped on PRS. | | | | | | | | |
